# Emerging Influenza D Virus Threat: What We Know so Far!

**DOI:** 10.3390/jcm8020192

**Published:** 2019-02-05

**Authors:** Kumari Asha, Binod Kumar

**Affiliations:** Department of Microbiology and Immunology, Chicago Medical School, Rosalind Franklin University of Medicine and Science, North Chicago, IL 60064, USA; asha.biotech@rediffmail.com

**Keywords:** influenza, influenza A virus (IAV), influenza B virus (IBV), influenza C virus (ICV), influenza D virus (IDV), emerging pathogen, epidemic, pandemic, respiratory illness, influenza-like illness

## Abstract

Influenza viruses, since time immemorial, have been the major respiratory pathogen known to infect a wide variety of animals, birds and reptiles with established lineages. They belong to the family *Orthomyxoviridae* and cause acute respiratory illness often during local outbreaks or seasonal epidemics and occasionally during pandemics. Recent studies have identified a new genus within the *Orthomyxoviridae* family. This newly identified pathogen, D/swine/Oklahoma/1334/2011 (D/OK), first identified in pigs with influenza-like illness was classified as the influenza D virus (IDV) which is distantly related to the previously characterized human influenza C virus. Several other back-to-back studies soon suggested cattle as the natural reservoir and possible involvement of IDV in the bovine respiratory disease complex was established. Not much is known about its likelihood to cause disease in humans, but it definitely poses a potential threat as an emerging pathogen in cattle-workers. Here, we review the evolution, epidemiology, virology and pathobiology of influenza D virus and the possibility of transmission among various hosts and potential to cause human disease.

## 1. Introduction

**Figure 1:**
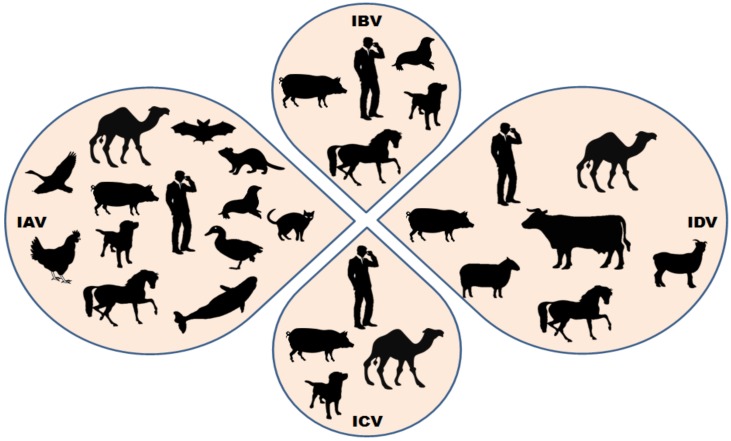
Summary of the wide host range of influenza viruses. Few host species are susceptible to infection by all the four known influenza viruses (influenza A virus—IAV, influenza B virus—IBV, influenza C virus—ICV and influenza D virus—IDV), while others are known to be infected by only specific influenza viruses. Cattles are the major known reservoir of influenza D virus; however, other small ruminants and humans have shown susceptibility to IDV infections in different conditions.

Influenza viruses belong to the family *Orthomyxoviridae*, which consists of four generas: Alphainfluenzavirus (Species: Influenza A virus (IAV)), Betainfluenzavirus (Species: Influenza B virus (IBV)), Gammainfluenzavirus (Species: Influenza C virus (ICV)) and Deltainfluenzavirus (Species: Influenza D virus (IDV)) [1,2,3]. They are enveloped viruses with segmented genome, comprising of single stranded negative sense RNA. Influenza viruses cause acute respiratory illness and of the four known types, the **influenza A virus** is a significant public health concern with highest rates of morbidity and fatality [4,5,6]. They have a wide host range (Figure 1) and often jumps the species barrier [7]. The emerging and reemerging threats due to IAVs are mainly due to two major events: Genetic drift and genetic shift [5,8,9,10,11]. *Genetic drift* is caused by point mutations in genes of the virus due to an error-prone RNA polymerase. Since the mutations take place in the genes encoding for the antibody binding sites, this event is responsible for (i) frequent influenza epidemics and (ii) the requirement of annual influenza vaccine formulations. *Genetic shift* is the result of genetic re-assortment between the genes of two or more different strains of influenza virus infecting the same cell in a host. This event occasionally generates novel combinations of the two major surface glycoproteins of the virus: Hemagglutinin (HA) and neuraminidase (NA). Since both genetic drift and genetic shift leads to changes in the genetic makeup of the virus, the existing immunity in the host may or may not provide complete protection and thus leads to multiple infections. Pigs, quails and bats have been reported to be the mixing vessels, because they co-express both SAα2,3-Gal and SAα2,6-Gal receptors required for binding with avian and human IAVs [12,13,14]. Genetic shift has created novel influenza virus strains with the ability to sustain efficient human-to-human transmission, and thus accountable for several influenza pandemics in the past [4,11]. A total of 18 novel HA (H1-H18) and 11 NA (N1-N11) have been identified till date, of which most of the possible combinations of 16 HA and 9 NA subtypes have been reported in water fowl which is the main reservoir of IAVs [15,16]. The recently identified H17-18 and N10-11 have not been reported in birds and have been found in bats [17,18]. To date, only 3 HA (H1, H2, and H3) and 2 NA (N1 and N2) subtypes have been reported to cause sustained, human-to-human transmission leading to influenza epidemics [19]. Apart from the human influenza strains, there are several low pathogenic avian influenza viruses (LPAI) and highly pathogenic avian influenza viruses (HPAI) that have been in circulation in wild aquatic birds and poultry thus posing a severe threat to public health. Few of the avian virus strains, such as the H5N1 and the newly emerged H7N9, have infected humans and thus are a significant cause of concern worldwide [20]. 

The **influenza B virus** was first identified in the year 1940 [21]. Unlike influenza A viruses that have a wide range of host, the IBVs almost exclusively infects humans with only a few reported cases in seals [22], horses [23], dogs [24] and pigs [25]. The IBVs do not have subtypes, but are categorized into two distinct lineages: B/Victoria/2/1987-like (B/Victoria-like) and B/Yamagata/16/1988-like (B/Yamagata-like) viruses that have been circulating worldwide since 1983 [26]. The influenza B viruses also cause annual epidemics, but due to the limitation of the host, they have never been known to be associated with any pandemic.

**Influenza C virus** was first isolated in the year 1947 [27], and similar to IBV, is known to cause relatively mild respiratory disease in humans [28]. ICV is widely distributed throughout the world majorly infecting children [29,30]. ICVs have also reported from pigs [31], feral dogs [32] and dromedary camels [33,34]. The relatively low prevalence of ICV and mild clinical outcomes has discouraged its inclusion in the routine virological screenings.

A novel influenza virus, recently classified as the **influenza D virus,** has been identified in several animals, such as swine, cattle and sheep from across the globe. IDVs are the latest member in the family *Orthomyxoviridae* with the potential of zoonotic and interspecies transmission. In this review, we have summarized the epidemiology, pathology, evolution and molecular biology of the novel influenza D virus that represents a potential threat to public health. 

## 2. Origin, Epidemiology and Pathogenesis of Influenza D Virus

Influenza viruses affect the human population in all age groups with an estimation of 5–10% in adults and 20–30% in children [35]. Of the approximately 3–5 million people who develop severe influenza infection, almost one million die annually [35]. Both influenza A and B viruses are known to cause annual epidemics and a strict surveillance program throughout the world is in action to monitor the slightest changes in their genome due to genetic drift or shift. IAV has also been associated with several pandemics in the past however, the IBV and ICV has never been the cause of any pandemic scenario owing to their limitation of host range and less frequent mutations reported till date [4,6,9]. The surveillance activities related to ICVs have not gained much importance compared to IAVs and IBVs as it has been associated with mild clinical conditions. The ICVs, apart from infecting humans, have also been reported to infect pigs in China (1981) [31] and the USA (2011) [36], dogs and camels [34]. The interspecies transmission of ICVs have been attributed to the genetic similarities that exist between the Chinese swine ICVs and human ICVs [37]. 

In the year 2011, an influenza C-like virus was isolated from pigs having a influenza-like illness in the USA. The newly identified influenza D virus shared approximately 50% homology with ICV and also lacked cross-reactivity between antibodies against IDV and human ICV, thus advocating the need of a separate genus in the family [36]. Hause et al. in their study further performed a detailed genetic, biological, and antigenic characterization of IDV and revealed that the novel IDV in cattle and swine were distinct from the human ICV [2]. They further revealed that bovines represent a natural reservoir for IDV as indicated by the widespread and high antibody titers against IDV and their ease of isolation from cattle [2]. Taking into account several of these differences to ICV, the International Committee on Taxonomy of Viruses (ICTV) classified Deltainfluenzavirus as a new genus of the family Orthomyxoviridae (Table 1).

Soon after the first isolation of IDV in Oklahoma, similar viruses were isolated from other part of the USA [38,39,40,41], as well as other countries, such as Mexico [38], China [42,43], Japan [44], France [45], Italy [46,47], Ireland [48] and Canada [40]. The IDV was also detected from equine populations and small ruminants, such as sheep and goat from the USA and Canada [40,49]. Although the study revealed a seroprevalence of 5.2% for sheep and 8.8% for goats suggesting that small ruminants could potentially be reservoirs of IDV and help transmit the virus to other domesticated animals [40], no IDV antibody was detected in the poultry [40]. Surveillance studies in China also reported that IDV was detected in goats and pigs [43]. The study showed that influenza viremia, as an indicator of disease severity, was observed in almost 30% of the cases with either acute infection or before the death of the host. An interesting observation was also revealed that IDV was detected in rectal swabs thereby raising the possibility of IDV to replicate within the intestinal tract similar to IAV and IBV [43]. In Japan, the virus has been in nationwide circulation in the cattle population for at least last seven years and the related study revealed that the rate of positivity of IDV infection tended to increase with the age of the animal and further indicated a horizontal transmission of the virus within a herd [50].

A surveillance study in Luxembourg demonstrated that the seroprevalence of IDV was observed to be around 80% in cattle in 2016 while it increased from 0% to 5.9% from 2012 to 2015 in the swine population [51]. The low seroprevalence of IDV in pigs suggests that the virus can infect and circulate in the pigs, but is not as wide spread as it is in cattle, thus suggesting that cattle are the major reservoir of this novel virus [51]. Yan et al., utilized the codon usage pattern to reveal evolutionary changes in the novel IDV’s hemagglutinin-esterase fusion (HEF) gene [52]. They further showed that although IDV could adapt to multiple hosts, especially cattle; however, swine exerted a stronger evolutionary pressure than the cattle [52].

Other reports revealed that newborn calves among the cattle had high levels of maternal antibodies against IDV which subsequently declined after six months of age showing both their exposure to the virus and vulnerability to IDV infection [39,41]. These studies also report that the IDV has been in circulation in the US cattle population much earlier than it was actually identified as a novel virus. Analyses were performed on the archived sera and it was observed that the circulation dates back to almost 2003 [39,41]. A similar study conducted on a large number of animals revealed that IDV has been in circulation in North and West Africa since at least 2012 in cattle and small ruminants [33]. The authors observed that the Dromedary camels in Kenya harbored influenza C or D virus antibodies suggesting a new host for IDV [33]. Surveillance on feral swine in the USA also showed a coinfection of IDV and IAV in the same host, thereby increasing the chances of reassortment and creation of novel virus strains [53]. The study also showed that the cattle shed IDV up to nine days post infection (dpi) while the swine shed the virus up to 5 dpi and attributed this difference to the predominance of virus in the upper respiratory tract in cattle while in the lower respiratory tract in swine [53]. Studies based on experimental infection in cattle showed that IDV was detected in both upper and lower respiratory tract however, the clinical signs and symptoms were mild [2,36,54]. Mitra et al. also suggested the possibility of coinfection by other respiratory viruses along with IDV to collectively cause the bovine respiratory disease (BRD) [38]. The cattle industry in the US and across the globe is majorly affected by BRD and it is estimated that in the US alone, the BRD leads to an annual loss of more than one billion dollars to the cattle industry [38]. BRD is often associated with multiple pathogens accounting for approximately 70–80% of the morbidity in the USA [38].

The IDV causes mild respiratory disease in experimentally infected cattle. Several studies have already been performed to analyze the pathology and mode of transmission of IDV in cattle and swine. In a study, the experimental results on swine indicated that the virus replicated in nasal turbinates and the virus shedding was detectable in nasal swabs with no clinical symptoms and lesions typical of influenza viruses. The study also revealed that IDV could be transmitted to naive animals by direct contact however; the replication of the virus was limited to the upper respiratory tract [36]. 

The study performed on cattle population revealed that IDV was detected in both upper and lower respiratory tract and observed transmission to other cattle in direct contact. The experimentally infected calves exhibited varying clinical signs and symptoms ranging from dry coughing and nasal discharges to lung auscultation score of 1 [54]. Few animals, however, had no clinical signs despite viral shedding [54]. The authors also examined the pathological consequences of IDV infection by performing histological analysis and observed that the tracheal inflammation was elevated in IDV-infected calves with multifocal areas of epithelial neutrophil infiltration and mild epithelial attenuation [54]. 

Mitra et al. performed viral metagenomic sequencing on the nasal swab samples obtained from cattle with acute BRD in Mexico and the USA, and revealed that the most commonly detected viruses were the bovine rhinitis A and B virus, and bovine coronavirus along with IDV [38]. Although this gives some evidence of IDV as one of the pathogens in BRD, more related studies are required to clearly understand the pathogenesis of IDV in context of BRD.

Unlike ICV that is typically cultured at 33 °C in experimental settings, the IDV shows optimal growth at both 33 °C and 37 °C further suggesting that IDV is not restricted to an elevated temperature for replication [36]. 

## 3. Evolution and Genome Structure of Influenza D Virus 

The IDV was first identified in pigs and has also been in circulation in cattle and several small ruminants across the world [2]. A recent study has shown that IDV has been circulating as two distinct genetic and antigenic lineages represented by D/swine/Oklahoma/1334/2011 (D/OK) and D/bovine/Oklahoma/660/2013 (D/660) [55]. The two lineages demonstrate frequent reassortment events with one another and also show antigenic cross-reactivity. Another interesting report revealed that IDV circulating in the bovine population of Japan formed an individual cluster that was distinct from strains reported from other countries, thus raising a possibility of unique evolution and pathology of IDV in Japan [56]. Phylogenetic analysis shows that the IDV clusters most closely with ICV suggesting a common ancestry for both viruses. Further the sequence comparisons of PB2, P3, NP, M and NS genes showed that IDV could be derived from human ICV [36]. Su et al. also suggests that the IDV clustered most closely with ICV based on the generation of a maximum-likelihood phylogenetic tree generated from the sequence of PB1 from influenza type A, B, C and D viruses [57]. Another phylogenetic analysis done based on all the seven genes of IDV showed that the IDV isolated from swine and bovine were both closely related and belonged to the D/swine/Oklahoma/1334/2011 cluster [46].

**Figure 2:**
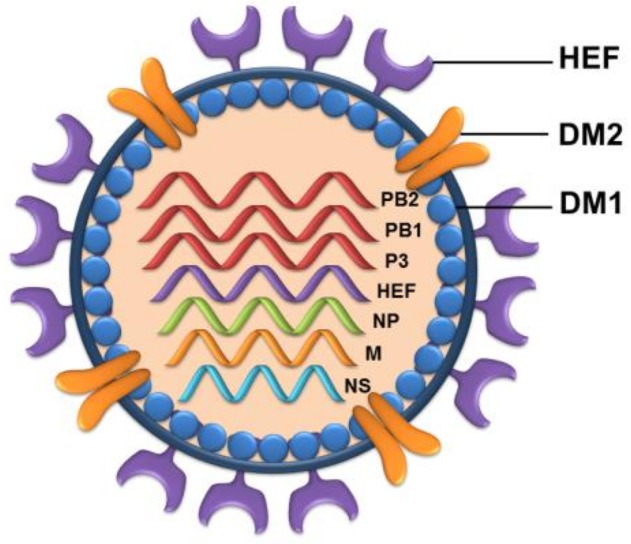
Schematic representation of influenza D virus. IDV is an enveloped virus with seven RNA segments. The virions are 80–120 nm in diameter. The glycoprotein hemagglutinin-esterase fusion (HEF) helps in viral entry into the host cells. The protein DM2 acts as the ion channel.

Unlike IAV and IBV that have eight negative-sense single-stranded viral RNA segments, the ICV and IDV have only seven segments that exist in the form of ribonucleoprotein complex (RNP) in association with nucleoproteins and an RNA-dependent RNA polymerase in virions (Figure 2).

Several studies in the past have revealed crucial functions performed by the individual genes of influenza A and B viruses [58], however very little of such studies have been performed in case of influenza C and D viruses. A scanning transmission electron microscopic tomography based study has revealed interesting information that although ICV and IDV have only seven segments, yet both the viruses package eight RNPs arranged in a “1 + 7” pattern regardless of the number of RNA segments in their genome [59].

The segmented genome of IDV encodes for nine proteins [54]. The longest three segments encode polymerases PB2, PB1, and P3 which are essential in replication and viral mRNA synthesis. The fourth segment encodes the glycoprotein hemagglutinin-esterase fusion (HEF) that shares extreme similarity between IDV and ICV in terms of structural folds and helps in viral entry. Also the IDV-HEF can accommodate diverse extended glycan moieties in its open receptor-binding cavity, thus providing an explanation for the broad cell tropism [60]. A recent study highlights the role of HEF glycoprotein of IDV in proving exceptional stability to high temperatures and acidity [61]. The authors revealed that IDV could withstand and retain its infectivity even at higher temperatures of 53 °C for as long as 120 min. Not only this, but the IDV was also observed to lose just 20% of the original infectivity when subjected to a low pH of 3.0 for 30 min as compared to IAV, IBV and ICV that were completely inactive at this low pH. This study demonstrates how IDV has evolved to utilize its proteins for survival in exceptional thermal and acidic conditions [61]. The fifth segment encodes the nucleoprotein (NP) which constitutes the viral ribonucleoprotein complex [62]. The sixth segment encodes the matrix proteins DM1 and DM2 that lines the viral membrane from inside and exhibits proton-channel activity respectively [4,63,64,65]. A recent study conducted on the *Xenopus laevis* oocytes, showed that the IDV-M2 protein was functionally similar to the ICV-M2 protein [66]. The last segment seven encodes nonstructural proteins NS1 and NEP that helps in neutralizing the cellular interferon response and mediates the nuclear export of RNPs respectively [67]. 

Homologies have been reported between IDV and ICV. A group of authors recently utilized the 5′ and 3′ RACE coupled with direct PCR sequencing to show that there is identity between the 3′ and 5′ non coding conservative regions of IDV and ICV with a single difference of adenine at position 5 at the 3′end of IDV while it is cytosine in ICV and a polymorphism at position 1 of the 3′ terminus [36]. The study further performed a ClustalW alignment of predicted PB1 amino acid sequences and revealed that IDV shared approximately 69–72% mean pairwise identity to ICV and 39%–41% identity to IAV and IBV. Similarly the HEF of IDV and ICV showed higher similarity; however it was lower for PB2, NS1, NP and M1 genes [36]. 

## 4. Influenza D Virus and Public Health Concerns

Studies show that IDV shares approximately 50% homology to human ICV and isolation of IDV from both swine and bovine indicates the possibility and potential of IDV to have successful transmission to other mammals, including humans [2]. A recent surveillance study conducted on the feral swine population in the USA revealed susceptibility to infections with both IAV and IDV [53]. They further observed that the seroprevalence rate of IDV in the IAV infected animals were much higher than the animals infected with only IDV, however this observation will need to be validated with a large statistically significant study covering more geographical areas of the world [53]. The coinfection in the feral swine poses a threat of carrying and transmitting the virus to other domestic swine and cattle. 

The studies related to the zoonotic potential of IDV is still in infancy and is unclear. The influenza viruses that cause zoonotic infections in humans have been shown to have poor transmissibility between ferrets compared to the viruses that cause epidemics and pandemics [68,69,70,71]. Recent studies show the ability of IDV to replicate and be transmitted among ferrets, which are the model for human influenza virus infection, and guinea pigs [72]. These studies highlight the potential of IDV to have the zoonotic transmission.

A surveillance study on a large number of human respiratory samples in Scotland revealed that IDV was not detected in any sample [73], however, another study performed to analyze the zoonotic potential of IDV in humans with occupational exposure to cattle, revealed a very high (94–97%) seroprevalence of IDV in humans exposed to cattle (calves), thus, indicating a possible emerging threat among cattle workers [74]. Although the serological analysis does suggest that IDV infects humans, yet in light of other studies that did not detect IDV in a large number of human samples, more epidemiological studies are required to come to a clear conclusion.

Although IDV genome structure is similar to ICV, they exhibit a broader cellular and host tropism than ICV [36]. The intensity and severity of the disease may become clearer with more number of relevant studies; however, the ability of influenza viruses to acquire frequent mutations makes IDV a potential threat to human health and warrants more comprehensive studies to understand its complexity and evolution. 

## 5. Strategies to Control IDV Infection and Management of Future Threat

Since the discovery of influenza viruses, annual epidemics and several pandemics have been recorded to claim numerous lives. With time, the influenza viruses have also shown remarkable evolutionary changes and their ability to adapt to a wide range of hosts. Influenza pandemics on one hand caused high morbidity and mortality and on the other hand taught several lessons that led to remarkable changes in the public health infrastructure. 

Surveillance activities against influenza viruses have been implemented worldwide to timely diagnose and prevent the spread of infection. A recent study developed and evaluated a real-time RT-PCR assay to detect the IDV in short time. The assay showed high sensitivity and specificity and can be used for diagnostics and as a confirmatory test for IDV infections [75]. Kishimoto et al., also described the development of one-run RT-PCR assay to not only detect IDV, but also 15 other respiratory pathogens, thus providing a quick and sensitive method to detect a wide range of respiratory viruses in the bovine respiratory disease complex [76]. In a most recent study, Henritzi et al. developed a tetraplex real-time RT-PCR assay for screening influenza virus types A, B, C and D simultaneously [77]. Moreno et al. focused on the development and validation of MAb-based competitive ELISA for the detection of antibodies against IDV which may be advantageous when compared to the traditional hemagglutination inhibition (HI) test [78]. 

Strategies like the bioaerosol surveillance at the human–animal interface may be advantageous in terms of low cost and less invasive sampling to detect novel influenza viruses [79]. Aerosol surveillance for respiratory viruses at an international airport in the USA from January to March 2018, revealed that few specimens were positive for IDV and adenovirus suggesting the feasibility of employing bioaerosol surveillance techniques in public transportation areas. This study, however, was performed on a very small sample size thus, a more elaborative analysis would be required to strengthen such findings [80]. 

Developments in the antiviral strategies and vaccine technology against influenza viruses have been in action to tackle disease outbreaks. Wan et al. in their recent study showed the protective efficacy of a DNA vaccine expressing consensus HEF protein against two lineages of IDV (D/OK and D/660) in guinea pigs. The study showed that the vaccinated animals showed significant titers of neutralizing antibodies against both lineages of IDV and were protected against the intranasal IDV challenge. Although studies in past have shown the potential of DNA vaccines against IAV [81], this study is claimed to be the first to show the effect of a DNA vaccine against IDV and thus a promising approach to manage infections [82]. 

Another report highlights the development of an inactivated influenza D virus vaccine that was found to be immunogenic and provided partial protection against IDV [83]. The immunohistochemistry showed that the vaccinated animals (calves) had significantly reduced the level of IDV titers in the respiratory epithelium of the nasal turbinates and trachea as compared to control animals [83]. Although some developments have been made to manage IDV infections, yet more extensive studies are required to evaluate the potential of existing and novel antiviral strategies to prevent any future IDV outbreak and potential zoonotic transmission to humans.

## 6. Conclusions

Influenza viruses have been the cause of significant concern to human and animal health worldwide. The virus not only causes morbidity and mortality, but its frequent infection also leads to socio-economic loss. Among the four genera, the influenza A viruses have been reported to be of significant concern owing to its ability to infect a wide range of hosts, and thus gaining the potential to jump the species barrier. Wild birds are the major reservoirs of IAVs [84,85,86,87] and approximately 105 different species of birds have been reported to harbor influenza A viruses [88,89]. Most of these carriers are almost asymptomatic [90,91] and can lead to the global spread of virus as infected birds may be able to fly longer distances while on migration [92,93].

Until recently, the influenza C viruses were known to exist as a single subtype with low rates of evolution, but the newly identified IDV (isolated from pigs in Oklahoma) representing influenza-like-illness shared approximately 50% homology to human ICV. In spite of the shared homology, the antibodies raised against IDV did not neutralize the human IAV, IBV or ICV in the serological analysis, and also the IDV showed a broad cell tropism unlike ICV, thus demanding a new genus in the family *Orthomyxoviridae*. Soon after the identification of IDV, several countries across the globe reported similar viruses circulating in both swine and cattle population. 

The novel zoonotic infections are often the result of pathogens crossing the species barrier and infecting novel host species. Although studies have shown that the IDV has not demonstrated a drastic antigenic change over the years, yet the unpredictability of influenza viruses make this type a potential health threat. IDV infections in small ruminants reported from various countries and feral swine population in the multiple states of the USA is an indication that further studies are urgently needed to clearly understand the status of IDV infections in other wild animals and the extent of interspecies transmission. In-depth studies are required to exactly assess the economic impact of IDV infections on the commercial livestock market. The mixed reports for IDV infections in humans further make it important to study the zoonotic potential of IDV, especially in people with occupational exposure to susceptible livestock. Since IDV shows potential to infect a wide range of host after IAVs, its zoonotic potential is a global concern. 

Although new studies have provided promising tools to manage IDV infections, yet more detailed investigations are needed to better understand this novel virus in terms of its epidemiological, pathological and biological characteristics specially when the capability of IDV to cause disease in humans have not been investigated in details and it’s not clear if the virus can sustain human-to-human transmission. 

Advancements in several approaches to managing influenza infections have led to quick and efficient control measures that were best seen during the 2009-H1N1 pandemic when the vaccines were developed in record time. Several strategies utilizing nucleic acid-based therapeutics have also been in action against several respiratory viruses, including human influenza viruses [94,95,96,97,98,99,100,101]. It would be interesting to have such strategies against IDV for timely management of the disease in cattle and other potential hosts.

As the influenza viruses continue to evolve, there is a need of joint efforts from medical doctors, scientists, veterinarians, agricultural industry and policy makers across the world to closely monitor the circulating influenza viruses and prevent any future outbreaks.

## Figures and Tables

**Table 1:** Revised classification of influenza viruses in the family *Orthomyxovirideae* by International Committee on Taxonomy of Viruses (ICTV).

Genus	Species	Number of Genomic Segments
Alphainfluenzavirus	Influenza A virus	8
Betainfluenzavirus	Influenza B virus	8
Gammainfluenzavirus	Influenza C virus	7
Deltainfluenzavirus	Influenza D virus	7
